# Relative contributions of upper-body muscular power and repeated sprint ability to 50-m freestyle swimming performance in competitive swimmers

**DOI:** 10.3389/fspor.2025.1751687

**Published:** 2026-01-15

**Authors:** Sofiene Amara, Anissa Bouassida, Roland van den Tillaar

**Affiliations:** 1 Higher Institute of Sport and Physical Education, University of Jendouba, Kef, Tunisia; 2 Department of Sports Science and Physical Education, Nord University, Levanger, Norway

**Keywords:** competitive athletes, fatigue index, force-velocity profiling, neuromuscular performance, performanceprediction

## Abstract

Upper-body muscular power and repeated sprint ability are recognized as important contributors to sprint swimming performance. However, the relative and combined predictive value of dry-land power measures and in-water repeated sprint ability for 50-m freestyle performance remains insufficiently understood. This study aimed to examine the relationships between upper-body muscular power, repeated sprint swimming variables (RSS), and 50-m freestyle performance in competitive swimmers. Thirty-six national-level male swimmers (age: 16.4 ± 0.3 years) participated in this cross-sectional study. Each swimmer completed: (1) an upper-body load–velocity assessment using the bench press on a Smith machine equipped with a linear position transducer to determine peak power; (2) an in-water repeated sprint test (8 × 15 m, 30 s rest) to determine fastest time, mean time, fatigue index, and total time; and (3) a maximal 50-m freestyle time trial. Pearson correlations revealed a very strong negative relationship between maximum muscular power and 50-m time (r = –0.86, *p* < 0.001), indicating that higher power was associated with faster swim performance. Among the RSS variables, fastest time (r = 0.83, *p* < 0.001), mean time (r = 0.78, *p* < 0.001), and total time (r = 0.78, *p* < 0.001) showed significant positive correlations with 50-m time, while fatigue index was not significantly related (r = 0.05, *p* = 0.78). The multiple regression model significantly predicted 50-m performance (R² = 0.86, *p* < 0.001), but only maximum muscular power emerged as an independent significant predictor (*β* = –0.027, *p* < 0.001). Upper-body muscular power was the strongest determinant of 50-m freestyle performance, explaining most of the variance in sprint time, while repeated sprint swimming variables reflect related in-water expressions of sprint capacity. Coaches should prioritize the development of dry-land power capacity alongside in-water anaerobic conditioning to enhance sprint performance. Combining load–velocity profiling and repeated sprint testing offers a complementary and practical framework for performance monitoring and individualized training prescription in competitive swimmers.

## 1 Introduction

Sprint swimming performance, particularly in the 50-meter freestyle event, represents the ultimate expression of alactic and lactic anaerobic capacities in competitive swimmers (1, 2). This event, typically lasting 21–28 s depending on athlete level, primarily demands immediate and short-term energy systems, with an estimated 65%–75% contribution from anaerobic metabolism (3). Within this specific physiological context, upper-body muscular power and the capacity to sustain a high velocity in the face of progressive lactate accumulation and neuromuscular fatigue emerge as critical performance determinants (4).

The scientific literature clearly established the fundamental role of upper-body muscular power in sprint performance. A recent meta-analysis by Crowley et al. (5) demonstrated significant correlations (r = −0.68 to −0.79) between bench press power measurements and 50-meter freestyle performance in competitive swimmers. More specifically, the force-velocity profile approach, validated by Samozino et al. (6), enables more refined characterization of athletes’ neuromuscular qualities. Previous studies have shown that elite swimmers exhibit maximum muscular power (Pmax) values 18%–25% higher than their lower-level counterparts (7), underscoring the importance of this physical quality for high-level performance. The transfer of this Pmax to effective propulsion in water constitutes a complex process that remains poorly quantified (8). This methodological limitation represents a significant gap in our understanding of sprint swimming performance.

Concurrently, the assessment of anaerobic capacities through ecological tests like the Repeated Sprint Swimming Test has gained popularity for its predictive validity. The work of Bishop et al. (9) found that mean time during an 8 × 15 m protocol correlated at r = −0.81 with 50-m performance time, while the fatigue index (calculated as the percentage decrement in time across sprints) showed a positive correlation (r = 0.62) with performance degradation at the end of the race. These results suggest that the capacity to maintain high velocity across multiple repetitions is a more relevant indicator of sprint performance than isolated maximum velocity (10). Nevertheless, understanding the interactive relationship between both approaches (i.e., dry-land muscular power and aquatic anaerobic capacities) and their relative contribution to swimming performance remains necessary.

However, despite these advances, several limitations persist in the current literature. First, few studies have simultaneously integrated dry-land power measurements and aquatic anaerobic capacities into a comprehensive predictive model. For instance, Garrido et al. (11) examined the effects of combined dry-land and aerobic training on performance but did not develop a statistical model to quantify their joint predictive power. Similarly, Loturco et al. (12) reported correlations between tethered swimming force, dry-land power, and sprint performance, yet their analysis stopped short of constructing a multivariate model to determine the relative contribution of each measure to competitive race times. Second, the interactive relationship between these different variables and their relative contribution to performance remains insufficiently quantified (13). Finally, existing studies often feature limited sample sizes or heterogeneous populations, compromising the generalizability of results. These gaps highlight the need for an integrative model, the distinct roles of neuromuscular power and metabolic capacity, into actionable insights for coaching practice. Such a framework would empower coaches to prioritize training interventions based on evidence of what matters most for sprint performance.

The present study therefore aimed to combine these different methodological approaches to develop an integrative model of sprint swimming performance. More specifically, our objectives were: (i) to quantify the relationships between upper-body muscular power, RSS variables, and 50-meter freestyle performance; (ii) to establish a multiple regression model predicting performance from these variables; (iii) to identify the relative contributions of each determinant to performance variance. Based on existing data, we hypothesize that the combination of dry-land power measurements and RSS variables would explain more than 75% of the variance in 50-meter freestyle performance.

## 2 Materials and methods

### 2.1 Study design

This study employed a cross-sectional correlational design to investigate the relationships between upper-body muscular power, anaerobic capacity, and sprint swimming performance. The research was conducted during the competitive season to ensure participants were in peak physical condition. All testing procedures were completed within a one-week period to minimize the effects of training variation on performance outcomes. Testing was performed over three separate sessions, with at least 48 h of rest between each session to ensure full recovery. The order of tests was as follows: Session 1: Force-velocity profile assessment; Session 2: Repeated sprint swimming (RSS) test; Session 3: 50-m freestyle time trial. All sessions were conducted at the same time of day (±1 h) for each participant. Participants were instructed to maintain their regular training diet and to avoid strenuous exercise for 24 h prior to each testing session. They were also asked to refrain from caffeine consumption on the day of testing.

### 2.2 Participants

Thirty-six male competitive swimmers (age = 16.4 ± 0.3 years, body mass = 68.4 ± 4.8 kg, height = 175.2 ± 5.7 cm) volunteered for this study. An *a priori* power analysis conducted using G*Power software (version 3.1.9.3) indicated that a sample size of 33 participants would be sufficient to detect a medium effect size (f² = 0.25) for a multiple linear regression model with three predictors, with a statistical power of 0.80 and an alpha level of 0.05. All participants were nationally ranked competitors specializing in sprint events (50-m and 100-m freestyle), possessing 8.3 ± 1.3 years of structured swimming training. At the time of the study, their in-water training volume averaged 45 ± 5 km per week, including high-intensity sprint and technique sessions. Their dry-land resistance training regimen consisted of three sessions per week, focused on strength and power development (e.g., Olympic lifts, ballistic throws, and maximal strength exercises) over the preceding two years, following a minimum of four years of general resistance training experience. Their personal best 50-meter freestyle times ranged from 26.56 to 28.12 s. After a comprehensive explanation of the study's objectives, procedures, potential risks, and benefits, written informed consent was obtained from all participants and their legal guardians. The study protocol was approved by the Institutional Ethics Committee of the Higher Institute of Sport and Physical Education of Kef, University of Jendouba, Tunisia (protocol code: 2025-31) and was conducted in strict accordance with the ethical principles of the Declaration of Helsinki.

### 2.3 Force-velocity profile assessment protocol

The dry-land testing session was conducted in a climate-controlled strength training facility, with environmental conditions maintained at 22.3 °C and 45% relative humidity, in alignment with established performance testing guidelines (14). The load–velocity profile was evaluated using a Smith machine bench press to ensure a strictly vertical bar path (6, 15). After completing a standardized warm-up (10 repetitions at 40% 1RM, 5 repetitions at 60% 1RM, and 3 repetitions at 70% 1RM), participants performed 4–5 single maximal-effort repetitions with progressively increasing external loads between 40% and 80% of 1RM. Barbell velocity was recorded using a linear position transducer (GymAware PowerTool, Kinetic Performance, Australia). The load–velocity relationship was analyzed via linear regression to determine key neuromuscular parameters (6, 15): theoretical maximal force (F₀), extrapolated as the force-intercept; theoretical maximal velocity (V₀), extrapolated as the velocity-intercept; and maximal muscular power (Pmax), calculated as (F₀ * V₀)/4. The parameter Pmax was used as the primary indicator of upper-body muscular power in subsequent analyses.

### 2.4 Repeated sprint swimming test

Anaerobic capacity was evaluated indirectly using a RSS test in a 25-meter swimming pool under controlled environmental conditions (water: 27.1 ± 0.5 °C; air: 25.9 ± 0.3 °C; humidity: 64 ± 5%), meeting competitive standards for thermal comfort and performance (16). The protocol commenced with a standardized 15-minute warm-up, structured as follows: 5 min of low-intensity front crawl swimming, followed by 5 min of technical drills (e.g., catch-up drill, fist swimming), and concluding with 5 min of progressive accelerations over 10–15 m (3–4 repetitions) to reach race-pace intensity. After 5 min of passive recovery, swimmers performed eight maximal 15-meter sprints from a push start in the water. The 15-meter distance was chosen to ensure a maximal sprint effort without the need for a turn. Swimmers were instructed to surface immediately and complete the entire distance at maximal intensity using front crawl stroke; underwater kicking was not permitted for ensuring standardization of the propulsion phase. After each sprint, swimmers returned to the starting wall at a low intensity. The total time between the start of one sprint and the start of the next (send-off time) was fixed at 40 s for all participants. This included the time to complete the 15-m sprint, the low-intensity return to the wall, and a standing rest to ensure exactly 30 s of passive recovery at the wall before the next start signal. All starts were synchronized using an auditory signal. The timekeeper initiated the stopwatch (SEIKO S120-4030, Tokyo, Japan) on the auditory start signal. Time was stopped when the swimmer's head passed the 15-meter mark. All timing was performed by the same experienced timekeeping specialist. From the recorded times, the following variables were calculated: the fastest 15-m sprint time (i.e., the shortest recorded time), the mean 15-m sprint time (total time/number of repetitions), the total time, and the fatigue index, calculated as: [(slowest time—fastest time)/fastest time] × 100. This protocol is based on established methods for assessing repeated-sprint ability (9, 17, 18).

### 2.5 50-m freestyle sprint test protocol

The 50-meter freestyle performance was assessed through a single maximal effort time-trial. The testing protocol began with a standardized 15-minute sport-specific warm-up, comprising 800–1,000 meters of progressive swimming, technical drills, and 3–4 accelerations over 15 meters. Following a 5-minute passive recovery period, participants performed one all-out 50-meter sprint from a dive start. Time was recorded by an experienced timekeeper using a handheld stopwatch (SEIKO S120-4030, Tokyo, Japan) (19, 20).

### 2.6 Statistical analyses

All statistical analyses were performed using IBM SPSS Statistics version 26.0 (IBM Corp., Armonk, NY, USA). Descriptive statistics, including means, standard deviations, and ranges, were calculated for all variables. The normality of data distribution was verified using Shapiro–Wilk tests, confirming the appropriateness of parametric analyses (21). Pearson correlation coefficients were computed to examine relationships between upper-body muscular power, RSS variables (mean time, fastest time, fatigue index, total time), and 50-m freestyle performance. Multiple linear regression analysis was conducted to determine the predictive power of independent variables on swimming performance. Regression assumptions were verified and met, including the absence of multicollinearity [all Variance Inflation Factor (VIF) values < 5], independence of errors (Durbin-Watson statistic ≈ 2), homoscedasticity, and normality of residuals. The significance level was set at *p* < 0.05 for all analyses.

## 3 Results

**Figure 1:**
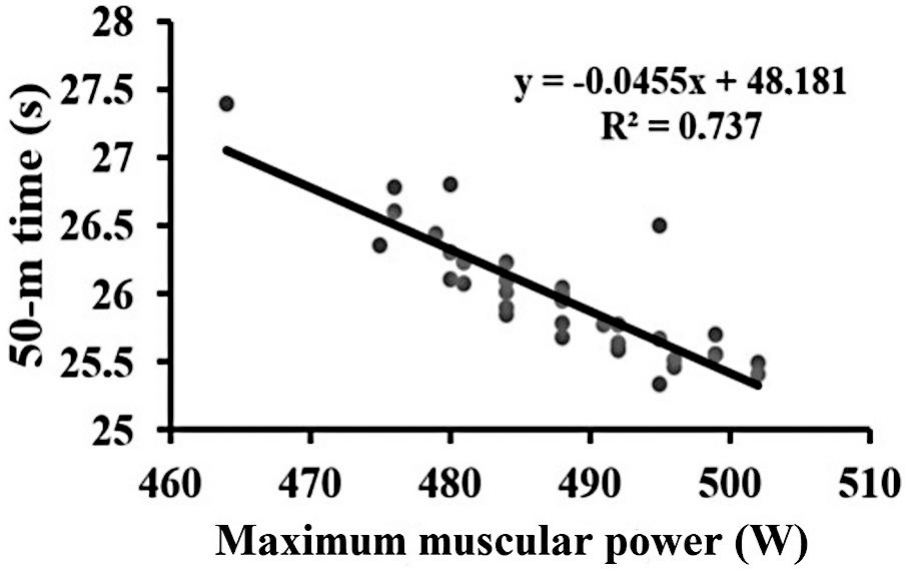
Scatter plot illustrating the relationship between upper-body maximum muscular power and 50-m freestyle performance time.

The statistical analysis revealed a strong relationship between maximum muscular power and swimming performance. Pearson's correlation showed a very strong negative association between power output and 50-m swimming time (r = –0.86, *p* < 0.001), indicating that swimmers with higher power levels achieved faster 50-m performance (Figure 1, Table 1).

**Table 1:** Correlation between 50-m swimming and independent variables.

Variable	Mean ± SD	r (with 50-m)	*p-value*
50-m swimming (s)	25.98 ± 0.46		
Pmax (W)	487.69 ± 8.59	−0.859	<0.000
Fastest time (s)	7.42 ± 0.17	0.832	<0.000
Mean time (s)	7.61 ± 0.18	0.779	<0.000
Fatigue index (%)	5.14 ± 1.01	0.049	0.776
Total time (s)	60.86 ± 1.45	0.779	<0.000

Pmax, maximum muscular power.

**Figure 2:**
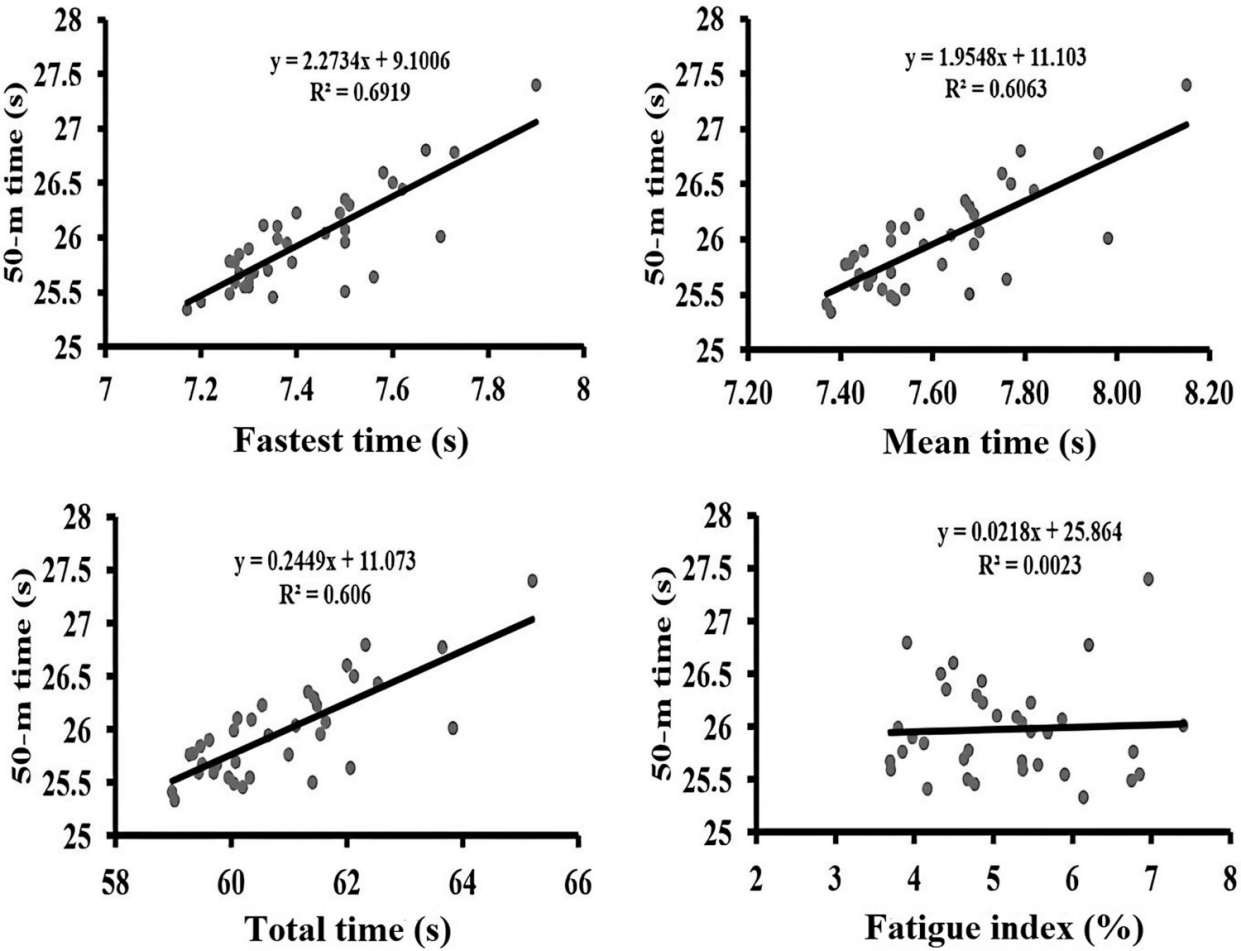
Scatter plots illustrating the relationships between repeated sprint swimming variables and 50-m freestyle performance time.

Correlations between the RSS variables and 50-m performance showed significant positive relationships for fastest time (r = 0.83, *p* < 0.001), mean time (r = 0.78, *p* < 0.001), and total time (r = 0.78, *p* < 0.001). Fatigue index did not show a significant correlation (r = 0.05, *p* = 0.78) (Figure 2, Table 1).

The multiple regression model including Pmax, mean and fastest time, fatigue index, and total time explained 86% of the variance in 50-m performance [R² = 0.862, adjusted R² = 0.844, F_(5,31)_ = 48.37, *p* < 0.001]. Among the predictors, only Pmax was a significant independent determinant (*β* = –0.027, *p* < 0.001). The other RSS variables did not significantly contribute to the prediction once Pmax was included in the model (Table 2).

**Table 2:** Multiple regression model for predicting the 50-m swimming.

Variable	Coefficient (β)	t	*p-value*
Constante	28.651	6.997	0.000
Pmax (W)	−0.027	−5.331	0.000
Fastest time (s)	3.789	1.097	0.281
Mean time (s)	−0.036	−0.698	0.490
Fatigue index (%)	0.036	0.308	0.760
Total time (s)	−0.290	−0.698	0.490

Pmax, maximum muscular power; R² = 0.862, adjusted R² = 0.844, F = 48.37, *p* (overall model) < 0.001.

## 4 Discussion

The main objective of this study was to examine the combined influence of upper-body muscular power and in-water RSS variables on 50-m freestyle performance in competitive swimmers. The results provide strong evidence that maximum muscular power is the primary determinant of sprint swimming performance, while repeated sprint swimming variables contribute secondarily. Together, these parameters explained 86% of the variance in 50-m performance, emphasizing the decisive role of neuromuscular power in short-distance swimming (22–24).

The very strong negative correlation between muscular power and 50-m time (r = –0.86) confirms the dominant contribution of upper-limb propulsion to sprint swimming performance. This observation aligns with biomechanical evidence showing that over 70% of total propulsive force in front crawl originates from upper-limb actions (8, 25, 26). Comparable studies by Morouço et al. (4) reported similar associations between dry-land power and sprint times (r = –0.82), supporting the ecological validity of load–velocity profiling for assessing sprint swimming potential. Unlike those investigations, which relied on single-load power assessments, the present study used a comprehensive profiling method, allowing more precise quantification of the mechanical capacity underlying performance (6, 15). The current regression coefficient (*β* = –0.027, *p* < 0.001) further demonstrates that improvements in muscular power translate directly into faster swimming, highlighting the practical importance of power-oriented dry-land training (7, 25).

Strong positive correlations were observed between RSS time variables and 50-m performance, particularly for the fastest 15-m sprint time (r = 0.83) and the mean 15-m sprint time (r = 0.78), indicating that swimmers who performed better in the repeated sprint swimming test also achieved faster 50-m race times, in agreement with previous studies linking repeated-sprint ability to anaerobic power and sprint performance (9, 27). However, despite these strong bivariate relationships, RSS variables did not remain a significant independent predictor in the multiple regression model, likely due to the substantial shared variance with dry-land muscular power measures. Both RSS performance and muscular power rely on common neuromuscular and anaerobic mechanisms, such as maximal force production, rate of force development, and phosphagen system efficiency (12, 13), and when entered simultaneously into the model, muscular power appears to capture a greater proportion of the variance in 50-m performance, thereby attenuating the unique predictive contribution of RSS variables.

Interestingly, the fatigue index from the repeated sprint test showed no significant relationship with 50-m performance (r = 0.05). This suggests that the specific capacity to resist fatigue across repeated bouts (as measured by the RSS decrement) is a distinct construct that may be less determinant for a single, brief maximal effort. While anaerobic lactic metabolism undoubtedly contributes to 50-m performance (3), our finding indicates that inter-individual differences in the rate of fatigue development during a repetitive test are not a key discriminator of 50-m time. This aligns with energy system models (28, 29) where the anaerobic alactic system provides the critical immediate power for the start and initial high velocity, and where a high anaerobic lactic capacity may support power maintenance without necessarily being reflected in a gross fatigue index from a different task structure. Thus, for the 50-m event, the ability to generate maximal instantaneous power (largely alactic) and to sustain a high power output (supported by lactic capacity) appears paramount, whereas the fatigue resistance specific to repeated efforts with short recovery may be a more specialized quality for different competitive demands.

The integration of dry-land and in-water measures offers a comprehensive view of performance determinants. While both types of measures are related to sprint success, the maximal muscular power derived from the force-velocity profiling method remained the only significant predictor after accounting for RSS variables. This suggests that this specific assessment captures the fundamental neuromuscular capacity (Pmax, F0, V0) underpinning overall sprint ability more precisely than simpler, single-load power measurements. This finding contrasts with earlier skepticism regarding the transfer of dry-land power to aquatic performance (30), demonstrating instead that dry-land power assessment provides essential information for individualized training prescription and talent identification (11).

This study is limited by its cross-sectional design, which prevents causal inference, and by its homogeneous sample of national-level male swimmers, reducing generalizability to other groups. Furthermore, the absence of direct metabolic measurements (e.g., blood lactate) prevents precise quantification of anaerobic metabolic involvement, requiring interpretation based on performance indicators alone. Moreover, technical, anthropometric, and start/turn parameters were not directly analyzed. Future research should employ longitudinal designs to confirm the causal link between increases in dry-land power and improvements in sprint performance (7). Including female swimmers, younger athletes, and different stroke specialists would enhance external validity. Integrating biomechanical and kinematic analyses with physiological testing could also clarify how muscular power improvements translate into propulsive efficiency in water (28, 31).

## 5 Conclusion

The findings of this study confirmed that maximum muscular power where assessed from the force-velocity profile in the bench press, is the primary determinant of 50-m freestyle performance, accounting for the majority of the observed variance, while repeated sprint swimming variables play a secondary role. These results emphasize the importance of prioritizing dry-land strength and neuromuscular power development, complemented by in-water training targeting sprint performance and anaerobic capacity. For coaches and sport scientists, integrating load–velocity profiling with repeated-sprint testing provides a reliable framework for performance monitoring and individualized training prescription in competitive swimmers.

## Data Availability

The datasets presented in this study can be found in online repositories. The names of the repository/repositories and accession number(s) can be found in the article/Supplementary Material.
